# Water Stress Is Differently Tolerated by Fusarium-Resistant and -Susceptible Chickpea Genotypes During Germination

**DOI:** 10.3390/life15071050

**Published:** 2025-06-30

**Authors:** Ümmühan Kaşıkcı Şimşek, Murat Dikilitas, Talap Talapov, Canan Can

**Affiliations:** 1 Faculty of Agriculture, Department of Agricultural Structures and Irrigation, Harran University, Şanlıurfa 63300, Turkey; 2 Faculty of Agriculture, Department of Plant Protection, Harran University, Şanlıurfa 63300, Turkey; m.dikilitas@gmail.com; 3 Faculty of Arts and Sciences, Department of Biology, Gaziantep University, Gaziantep 27410, Turkey; talapov10@gmail.com (T.T.); ayselcan1938@gmail.com (C.C.)

**Keywords:** chickpea, drought stress, fusarium wilt, combined stress, germination

## Abstract

Chickpea is a legume that grows in most parts of the world. It is negatively affected by abiotic and biotic factors like drought and fungal diseases, respectively. One of the most important soil-borne pathogens affecting chickpeas is *Fusarium oxysporum* f.sp. *ciceris* (*Foc*). Its population dynamics in the soil are affected by fluctuations in soil water content and host characteristics. For the last three decades, drought has been common in most areas of the world due to global warming. Drought stress decreases the quality and quantity of the chickpeas, particularly where soil-borne pathogens are the main stress factor for plants. The use of both drought-tolerant and disease-resistant cultivars may be the only option for cost-effective yield production. In this study, we screened the seeds of twelve chickpea genotypes WR-315, JG-62, C-104, JG-74, CPS-1, BG-212, ANNIGERI, CHAFFA, BG-215, UC-27, ILC-82, and K-850 for drought tolerance at increasing polyethylene glycol (PEG) concentrations (0-, 5-, 7.5-, 10-, 15-, 20-, 25-, 30- and 50%) to create drought stress conditions at different severities. The performances of genotypes that were previously tested in *Foc* resistance/susceptibility studies were assessed in terms of percentage of germination, radicle and hypocotyl length, germination energy, germination rate index, mean germination time, and vigor index in drought conditions. We determined the genotypes of C-104, CPS-1, and WR-315 as drought-susceptible, moderately drought-tolerant, and drought-tolerant, respectively. We then elucidated the stress levels of selected genotypes (20-day-old seedlings) at 0–15% PEG conditions via measuring proline and malondialdehyde (MDA) contents. Our findings showed that genotypes that were resistant to *Foc* also exhibited drought tolerance. The responses of chickpea genotypes infected with *Foc* under drought conditions are the next step to assess the combined stress on chickpea genotypes.

## 1. Introduction

Chickpea (*Cicer arietinum* L.) is an important legume plant that is cultivated in many parts of the world. It is a grain legume with a high protein content [1]. The global demand for chickpeas has grown rapidly as more people become aware of the health advantages of chickpeas as a vegetarian protein source [2]. Furthermore, it promotes nitrogen fixation in the soil and minimizes fertilizer inputs [3]. According to physiological and genetic properties, chickpea plants are split into two main types called “desi (microsperma)” and “kabuli (macrosperma)”. The desi type is identified by its pink flowers and anthocyanin accumulation in the stem with a thick seed coat. In contrast to desi, the kabuli type has white flowers, and its stems are devoid of anthocyanin pigmentation. Their seeds have a thin seed coat with white or beige colors [4]. Desi type is generally produced in Southeast Asia and South Africa, and occupies 80–85% of the global chickpea area, whereas the kabuli type is mostly grown in West Asia, North Africa, North America, and Europe [1].

Water shortage has been a serious obstacle to agricultural production throughout the world in the last decade [5]. Drought is one of the most important abiotic stresses, resulting in almost 50% of crop losses worldwide annually [6]. Drought stress can significantly impact the life cycles of cultivated plants, leading to substantial production losses. The extent of these losses depends on several factors, such as the duration and severity of the stress and the growth stages of the plants.

Water sources are decreasing globally. The selection of drought-tolerant varieties is required for many crop species [7]. At early growth stages of plants, the development is delayed, and losses in crop yield are inevitable [8]. Previous studies have demonstrated that the seedling viability index, which correlates well with seed germination rates, serves as a valuable indicator for assessing drought tolerance at the start of the growing season [9]. Recently, osmotic solutions such as PEG (polyethylene glycol) and mannitol have been used to evaluate drought stress tolerance for many crop species [10]. PEG is a chemical substance with a high molecular weight and causes water stress in plants by increasing osmotic pressure without interfering with cell metabolism [11]. The efficacy of PEG in inducing drought stress during the germination and seedling growth stages of chickpeas was justified in previous works [5,12].

*Fusarium oxysporum*, located in the Ascomycota phylum, Hypocreales order, comprises soil-borne pathogens that can maintain their viability in the soil for many years through the clamydospores they produce. Pathogenic forms of this fungus penetrate the root system and colonize the root cortex [13]. After penetrating the plant, the pathogen spreads to the xylem in the vascular bundles and propagates. Because of pathogen growth and due to the formation of tyloses in the xylem, the tissue becomes partly clogged, and water and solute movement become impaired [14]. Consequently, chlorosis and wilting in the top parts and root rot become characteristic symptoms over time. Plant death in sensitive varieties is inevitable [15]. In recent years, wilting and chlorosis have also been attributed to the hormone and enzyme production of pathogenic fungi [16,17]. Since ethylene and abscisic acid (ABA) hormones are involved in water stress, drought, and *Foc* combinations are highly likely to be involved in these hormones, which are characterized by wilting and chlorosis.

*F. oxysporum* is a large cryptic species that exhibits host specialization and is divided into *formae speciales* groups. Fusarium wilt agent in chickpea has been identified as *F. oxysporum* f. sp. *ciceris* (Padwick) Snyder and Hansen (*Foc*), and there are different pathotypes (yellowing and wilting) and races (0, 1A, 1B/C, 2, 3, 4, 5, and 6) of the agent depending on the chickpea genotypes [18,19]. The yellowing pathotype induces progressive foliar yellowing with vascular discoloration, while the wilting pathotype causes severe and fast chlorosis, flaccidity, and vascular discoloration [20,21]. Based on their reactions to a set of chickpea differential lines, eight physiological races of *Foc* (0, 1A, 1B/C, 2, 3, 4, 5, and 6) have been identified [18,22,23].

Drought stress not only affects the conditions of crop plants but also impacts the pathogens in the soil. Pathogen propagation, spread, and infection capacity could be affected by drought or water stress, depending on the duration and severity of drought stress. The important issue here is the survival of plant pathogens. For example, conidial production and pathogenicity of *Fusarium* and *Rhizoctonia* spp. were either increased or maintained under drought conditions [24,25]. It is clear that the maintenance of sporulation and mycelial growth of fungi would enable pathogens to develop in host tissues and maintain pathogenicity. Therefore, drought and pathogen interactions have been the focus of attention worldwide recently [26]. Drought stress has been more common and intense due to an increase in air and soil temperature, especially in spring and summer, along with the limited precipitation in semi-arid and arid areas. It is, therefore, expected that the impact of a soil-borne pathogen, e.g., *Fusarium* spp. would further increase if drought stress cannot prevent the pathogen from sporulating and developing under such conditions. Thus far, many studies have shown that drought stress and pathogen infection have additive stress effects on crop plants.

In this study, the previously tested chickpea genotypes for Fusarium wilt resistance were assessed in drought-simulated conditions to determine if there is a connection between drought tolerance and Fusarium wilt resistance in chickpea genotypes. Fusarium wilt tolerance under drought stress conditions will also be revealed in our next study to elucidate if the pathogen is a potential danger under drought stress conditions. Therefore, it would be more appropriate to investigate the effects of combined drought-pathogen stress and resistant-tolerant genotypes.

## 2. Materials and Methods

### 2.1. Plant Materials

This study was conducted at Harran University (Şanlıurfa-Türkiye), Faculty of Agriculture, Phytopathology laboratories. Chickpea seeds of various genotypes exhibiting different responses to *Fusarium oxysporum* f.sp. *ciceris* (WR-315, JG-62, C-104, JG-74, CPS-1, BG-212, ANNIGERI, CHAFFA, BG-215, UC-27, ILC-82, K-850) were kindly provided by Prof. Abdullah Kahraman, Department of Field Crops, Harran University.

### 2.2. Treatments and Experimental Materials

Chickpea seeds of uniform size and shape selected for each variety were sterilized with 1% NaOCl solution for approximately 2 min, followed by immersion in 70% ethanol for 1 min and rinsing with distilled water three times. To simulate drought stress conditions, the seeds were placed on filter paper in Petri dishes and treated with 20 mL of PEG6000 solutions (0-, 5-, 7.5-, 10-, 15-, 20-, 25-, 30-, and 50%). The measurement of percentage PEG6000 was also expressed as megapascal (MPa) in water potential (WP, ψ_w_). According to Michel and Kaufmann [27]; 0-, 5-, 7.5-, 10-, 15-, 20-, 25-, 30-, and 50% PEG6000 were expressed as 0, −0.05, −0.075, −0.15, 0.22-, 0.5- 1.35-, 1.0-, and 2.7- MPa. The measurements were also confirmed with Chemlab Scientific Product Ltd. 5500 Vapor Pressure Osmometer, London, UK. The plates were sealed with parafilm to prevent evaporation before being placed in an incubator (Nüve ES 120, İstanbul, Türkiye) for germination. Each Petri dish containing five seeds from each treatment was accepted as a replicate, and a total of four replicates were employed for each treatment (concentration) of each genotype. Germination tests were conducted under dark conditions at a temperature of 25 ± 2 °C and 65% relative humidity.

### 2.3. Measurement of the Germination Parameters

Chickpea genotypes were assessed in terms of % germination (Equation (1)), germination energy (GE, Equation (2)), vigor index (VI, Equation (3)), germination rate index (GRI, Equation (4)) (Ullah et al., 2022), and mean germination time (MGT, Equation (5)) [28,29,30]. The seeds were considered germinated when the radicle length reached 2 mm. Following the completion of germination tests, inhibitory concentrations at 50% (IC_50_) were calculated for each genotype. On the eighth day, hypocotyl and radicle lengths were also measured and recorded.

(1) $$Germination\;Percentage\left(\%\right)=\frac{Number\;of\;germinated\;seeds}{Total\;number\;of\;seeds}\times 100\;$$

(2) $$Germination\;Energy=\frac{Number\;of\;germinated\;seeds\;on\;the\;fifth\;day}{Total\;number\;of\;seeds}\times 100$$

(3) $$Vigor\;Index\;(VI)=\frac{Mean\;radicle\;length+Mean\;hypocotyl\;length}{Germination\;percentage}$$

VI: Vigor index is a significant parameter to evaluate overall health and robustness of germinating seeds using germination percentage and seedling growth parameters.

(4) $$Germination\;Rate\;Index\;(GRI)=\frac{G1}{D1}+\frac{G2}{D2}+\ldots +\frac{Gx}{Dx}$$

GRI: Germination rate index is a germination parameter reflecting how quickly and uniformly seeds germinate over a given time period.

(5) $$Mean\;Germination\;Time\;\left(MGT\right)=\frac{\left(\left(G1\times 1\right)+\left(G2\times 2\right)+\ldots +\left(Gn\times n\right)\right)}{Total\;number\;of\;seeds\;germinated\;after\;8\;days}$$

*G*: Count of germinated seeds recorded on the respective day

*D*: Day

IC_50_ values were calculated for each variety [31,32].

IC_50_: Inhibitory concentration that prevents 50% of germinating seeds. This criterion was used as a threshold level to decide the breaking point that disturbs the physiological, biochemical, and molecular mechanisms in germinating seeds.

Y=ax+b Typical linear regression formula

$Y=ax^{2}+bx+c$ A typical quadratic analysis formula

$${IC}_{50}=\frac{-b-\sqrt{b^{2}-4a(c-50)}}{2a}$$

Following germination tests, we selected three chickpea genotypes as drought tolerant (WR-315), moderately drought tolerant (CPS-1), and drought susceptible (C-104) according to their responses to PEG. The seeds of genotypes were tested again at 0-, 5-, 10-, and 15% PEG6000 conditions, and their biochemical responses, such as proline and MDA at the seedling stages, were determined. For this, the selected seeds based on germination parameters and IC_50_ performance were arranged in three replicate groups of 20 seeds for each variety and placed on moist filter paper. The seeds were then covered with another layer of moist filter paper and rolled into cylinders. For the control group (0% PEG6000), 200 mL of water was added to the rolls placed vertically in beakers, while the drought groups received 20 mL of water containing 5-, 10-, and 15% PEG6000, respectively. The rolls were incubated in an incubator, and the seedlings were grown for a period of eight days at a temperature of 25 ± 2 °C. For proline and MDA analysis, the seedlings were allowed to grow for an additional 12 days after 8 days of germination, making them ready for the analysis. A total of 20 plants from three replicates of each treatment were mixed, and 0.5 g was used for the analyses.

### 2.4. Measurement of the Leaf Proline Content

Proline contents of the seedlings under various drought stress conditions were determined according to [33] with minor modifications. A reaction mixture was prepared by dissolving 1.25 g of ninhydrin in 30 mL of glacial acetic acid and 20 mL of 6 mol L^−1^ phosphoric acid. A fresh leaf sample (0.5 g) was homogenized in liquid nitrogen and solubilized in 10 mL of 3% sulfosalicylic acid. Proline was extracted from the leaves by homogenizing them in 3% sulfosalicylic acid, and the resulting supernatant was combined with an equal volume of glacial acetic acid and acidic ninhydrin. The mixture was heated at 100 °C for 30 min and then allowed to cool, after which 5 mL of cold toluene was added. The toluene phase was measured at 520 nm with a spectrophotometer device (Thermo Scientific Multiskan Skyhigh, Waltham, MA, USA), and the concentration of proline was determined using a calibration curve and expressed as μmol proline g^−1^ fresh weight.

### 2.5. Measurement of Leaf Lipid Peroxidation (MDA) Levels

Malondialdehyde content (MDA), a product of lipid peroxidation, was measured using a modified version of the [34] method. A fresh leaf sample (0.5 g) was homogenized in 10 mL of 0.1% trichloroacetic acid (TCA) and then centrifuged at 10,000× *g* for 5 min. After the following centrifugation, 4 mL of 20% TCA containing 5% thiobarbituric acid (TBA) was added to 1 mL of the extract. The resulting mixture was incubated at 95 °C for 30 min. Then, an ice bath was used to quickly cool it. After centrifugation at 10,000× *g* for 10 min, 300 μL supernatant was taken and placed into plates, and the absorbance readings were made in the spectrophotometer (Thermo Scientific Multiskan Skyhigh, Waltham, MA, USA) at 532 and 600 nm. The results were expressed as nmol g^−1^ FW.

$$MDA\;({nmol\;g}^{-1}FW)=\frac{Extract\;volume\;(ml)\times \left\lceil {(A}_{532}-A_{600})/155\;{mM}^{-1}{cm}^{-1}\right\rceil }{Sample\;quantity(g)}\times 10^{3}$$

### 2.6. Statistical Analyses

IC_50_ values of chickpea genotypes, based on the relationship between germination values and drought doses, were calculated using Probit analysis. The IC_50_ values were calculated for each genotype. Analysis of variance and Duncan’s Multiple Range Tests at a significance level of *p* < 0.05. All statistical analyses were conducted using IBM SPSS Statistics v.26 (IBM Corporation, New York, NY, USA). Results were expressed as mean ± SE.

## 3. Results

### 3.1. Results of Germination Parameters

The germination responses of various chickpea genotypes to different concentrations of polyethylene glycol (PEG6000) are presented in Table 1. Increases in PEG6000 concentration led to decreases in germination in all genotypes of chickpea. The decreases in germination were prevalent between 20% to 50% of PEG6000. According to the IC_50_ calculation, WR-315, CPS-1, and C-104 genotypes were determined as drought-tolerant, moderately drought-tolerant, and drought-sensitive cultivars, respectively. A short-term drought stress induced by PEG6000 led to decreased germination of chickpea genotypes at 15% PEG6000 and higher doses. A significant reduction in germination rates with increasing drought stress was evident in all genotypes. Genotype C-104 showed the least germination capacity among all chickpea genotypes. At 10% PEG6000, the C-104 genotype did not germinate at all. WR-315 showed the best germination performance, which was able to germinate up to 20% PEG6000 concentration with an 85% germination rate. The IC_50_ value of WR-315 was calculated as 13.93% PEG6000. The rest of the genotypes showed a remarkable decline at 15% PEG6000 germination. None of the genotypes could germinate at 30% PEG6000 (Table 1). Germination percentage was higher at 5–7.5% PEG6000 than that of the control group. PEG6000 at low doses might act as a priming agent, improving germination by maintaining plasma membrane integrity. A delay in mean germination time and a decrease in vigor index were observed with increasing PEG6000 doses (Table 1). IC_50_ values ranged from 6.95 (UC-27, kabuli type) to 17.76 (BG-212, desi type) (Table 2). Desi-type chickpea genotypes were generally more drought-tolerant than kabuli-type genotypes.

Since almost all parameters showed similar trends to that of germination percentage, we chose the percentage germination parameter and calculated IC_50_ values for each cultivar and used this criterion for the selection of cultivars in terms of drought tolerance.

### 3.2. Selection of Tolerant/Moderately Tolerant and Susceptible Genotypes on the Basis of Their IC_50_ Values

The IC_50_ values followed by PEG-induced water stress varied notably among the chickpea genotypes (Table 2). Higher IC_50_ values reflect greater drought tolerance, as a higher PEG concentration is required to reduce germination by half.

Increases in PEG6000 concentration led to decreases in germination in all genotypes of chickpea. The decreases in germination were prevalent between twenty and fifty percent of PEG. According to the IC_50_ calculation, we selected three genotypes categorized as tolerant (WR-315), moderately tolerant (CPS-1), and sensitive (C-104), respectively. The drought responses of all genotypes and their respective IC_50_ values are presented in Table 2 along with their pathological responses. The pathological responses of those genotypes were already determined previously [35,36].

Short-term drought stress induced by PEG6000 markedly inhibited germination in chickpea genotypes, with complete germination failure observed at 30% PEG6000 concentration (Table 1); among the tested genotypes, WR-315 demonstrated the highest drought tolerance, while C-104 exhibited extreme sensitivity, failing to germinate even at 10% PEG6000. GRI and GE measurements were in line with the percentage germination in all genotypes. When the impact of drought stress became more severe, MGT increased considerably (Table 1). All genotypes were not able to produce hypocotyl and radicle at 15% and higher concentrations of PEG during the germination period.

The statistical calculation was made in each genotype, and lettering was made on the basis of germination in each treatment (*p* < 0.05). Twenty seedling plants were employed for each treatment per genotype.

**Figure 1:**
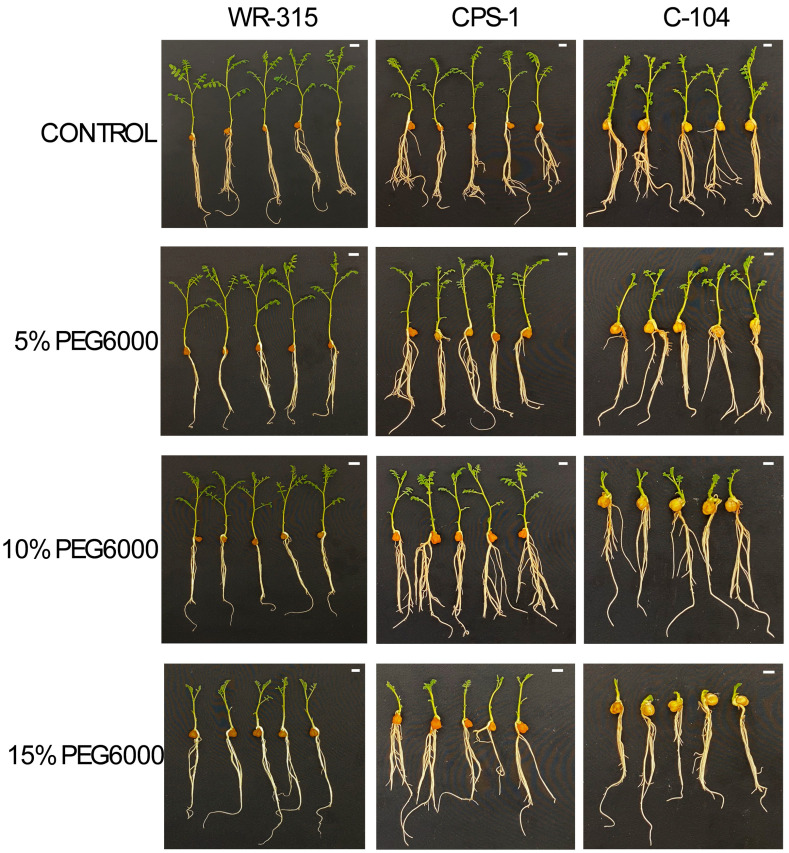
Post-germination stages of the drought-tolerant (WR-315), moderate drought-tolerant (CPS-1), and drought-susceptible (C-104) genotypes to water stress under 0-, 5-, 10-, and 15% of PEG6000 concentrations. The white bar in the upper right corner of each photograph represents 1 cm.

**Figure 2:**
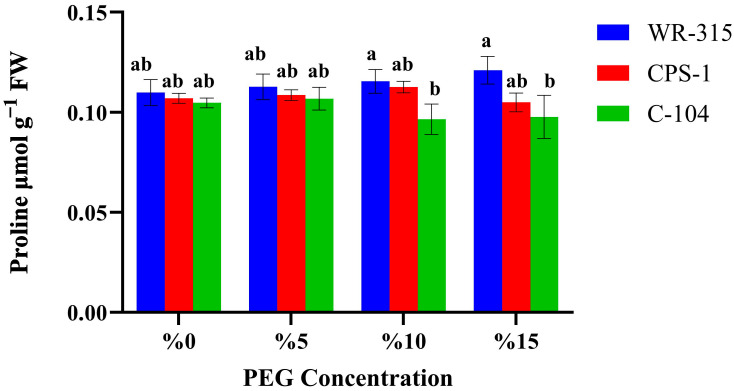
Proline contents of WR-315, CPS-1, and C-104 chickpea genotypes at different PEG6000 doses. Different letters in bars show significant differences at the *p* < 0.05 level; results were expressed as mean ± SE of bars.

**Figure 3:**
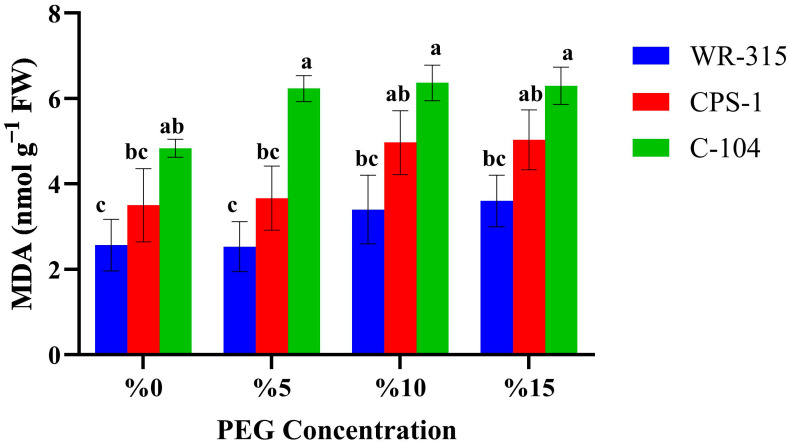
MDA contents of WR-315, CPS-1, and C-104 chickpea genotypes at different PEG6000 doses. Different letters in bars show significant differences at the *p* < 0.05 level; results were expressed as mean ± SE of bars.

The most drought-tolerant genotype was WR-315, which showed greater performance at 25% PEG6000 than the other genotypes. The remaining genotypes were classified accordingly. In general, desi-type chickpea genotypes were more drought-tolerant than kabuli-type genotypes (Table 2). The IC_50_ values ranged from 6.95 (UC-27, a kabuli type) to 17.76 (BG-212, a desi type) percentage (Table 2). The findings are in line with [37]. They emphasized that desi genotypes had better germination, trehalose, and sugar values than kabuli genotypes under drought conditions. On the basis of IC_50_ values of germinating seeds of WR-315, CPS-1, and C-104 genotypes were further evaluated in terms of biochemical responses at various drought conditions, Figure 1, Figure 2 and Figure 3. Please note that the stem length of C-104 gets shorter as the impact of drought stress increases. We did not observe similar physiological responses in the tolerant genotype of chickpea, WR-315. At later stages, C-104 genotypes dried out very quickly. We noticed that the selected drought-tolerant (WR-315), moderately drought-tolerant (CPS-1), and drought-susceptible (C-104) chickpea genotypes were also disease-resistant, moderately disease-resistant, and disease-susceptible genotypes, respectively, to *Foc* [35,36]. Races 2, 4, and 5 of *Foc* are determinant characteristics for the selection or screening of races against chickpea genotypes.

When selected genotypes were visually examined, WR-315 did not exhibit significant changes in terms of hypocotyl and radicle length across the PEG doses. The genotype was able to grow efficiently. The moderately tolerant genotype also showed significant growth. The drought-susceptible genotype, C-104, was also able to grow in drought conditions; however, its root structure showed weakness in terms of lateral root formation.

### 3.3. Biochemical Responses of Selected Genotypes

We determined the biochemical responses of genotypes to evaluate drought pathways in drought-, moderately drought-tolerant, and susceptible genotypes. Thus, proline accumulation in chickpea genotypes WR-315, CPS-1, and C-104 was assessed under increasing concentrations of PEG6000 (Figure 2). In general, proline content increases with the increase in drought stress doses, indicating a stress-responsive accumulation pattern.

Among the genotypes, WR-315 consistently showed higher proline levels across all PEG treatments, with statistically significant increases at 10 and 15% PEG concentrations compared to C-104. CPS-1 exhibited an intermediate response, with proline levels comparable to WR-315 at 5 and 10% PEG, but lower at 15% PEG. In contrast, C-104 showed the lowest proline accumulation at higher PEG concentrations, particularly at 10 and 15%, where values were significantly lower than those of WR-315.

These results suggest that WR-315 has a stronger osmoprotective response under drought stress, potentially contributing to its higher stress tolerance. The relatively lower proline accumulation in C-104 may partly explain its greater sensitivity to PEG-induced osmotic stress, as also reflected in its lower IC_50_ value and germination performance under similar conditions.

Malondialdehyde (MDA) content, an indicator of lipid peroxidation and oxidative membrane damage, was measured in WR-315, CPS-1, and C-104 chickpea genotypes under increasing PEG6000 concentrations (Figure 3). Overall, MDA levels increased with rising PEG concentrations in all genotypes, indicating enhanced oxidative stress under drought conditions.

Among three genotypes, C-104 consistently exhibited the highest MDA content at all PEG concentrations, with significant increases observed starting from 5% PEG. Notably, MDA levels in C-104 peaked at around 7 nmol g^−1^ FW under 10 and 15% PEG, significantly higher than both CPS-1 and WR-315.

In contrast, WR-315 maintained the lowest MDA levels across all treatments, including under severe stress (15% PEG), suggesting a more effective oxidative stress mitigation mechanism. CPS-1 displayed intermediate MDA accumulation, with moderate increases in response to PEG stress.

These results are consistent with general drought tolerance patterns, where WR-315 showed superior tolerance, potentially due to better protection against membrane lipid peroxidation. The elevated MDA levels in C-104 suggest that membrane damage correlates with its lower IC_50_ value and reduced germination under PEG-induced drought.

## 4. Discussion

Drought is widely recognized as one of the most significant sources of environmental stress because of its extensive impact on agricultural areas and its adverse effects on almost all plant species [38]. Plant responses to drought stress are extremely complicated. Drought stress induces morphological, physiological, biochemical, and molecular changes and significantly alters those metabolic pathways [39].

The involvement of the pathogen makes these responses more complex and affects almost all metabolic pathways. In this study, the responses of 12 chickpea genotypes with various responses to *Fusarium oxysporum* f. sp. *ciceris* (*Foc*) were tested under different water stress conditions. The percentage of PEG6000 values was also converted into megapascal (MPa) values to be assessed with other publications.

Similarly, Ahmad et al. [40] reported that drought conditions simulated by PEG6000 affected germination parameters of *Stevia rebudiana* under in vitro conditions. Adetunji et al. [41] stated that PEG could act as a priming agent and maintain plasma membrane integrity by regulating and extending the hydration period in seeds. At high doses, it results in osmotic stress and thus establishes drought conditions in organisms; therefore, PEG is a good selection to create drought stress without interfering other metabolites due to high molecular weight characteristics in cells. It is non-toxic, non-permeable, and mimics the moisture of dry soil. Since the creation and maintenance of uniform water potential in the field are challenging, an in vitro screening method using osmoticum at different concentrations enabled us to evaluate drought stress at early growth stages in various genotypes. Therefore, in vitro selection allowed us to examine the biochemical responses of seedlings at early stages uniformly. Increases in PEG6000 concentration resulted in a significant decline in germination percentage and extended germination period. Similar findings were reported by Himaja et al. [42] and Abdallah Abderemane et al. [43], who stated that germination under water-stressed conditions via PEG was a valuable trait for identifying tolerant genotypes and reflected in those created under field conditions. They also concluded that the most promising genotypes were of the Desi type, which aligns with our findings. PEG disrupts the uptake of water, creating physiological drought, which is characterised by low water potential in the soil. Therefore, PEG could simulate low rainfall conditions in the soil during seed germination. Since germination is a critical stage in establishing root and stem structure, it is essential to identify tolerant varieties at this stage. Performing metabolomics and genomics at the initial stage, followed by the establishment of drought and pathogen stress conditions, would enable us to avoid incurring a significant amount of time, effort, and financial costs. Under drought stress, accumulation of compatible solutes is most prevalent for reducing osmotic stress as they play significant roles for ion homeostasis and membrane integrity [44,45]. Accumulation of compatible solutes may also play crucial roles in the protection of proteins and enzymes from denaturation [46,47]. Proline, one of the widely studied compatible solutes, keeps water molecules due to its hydrophilic characteristics [48,49]. Proline accumulation is, in general, positively correlated with stress tolerance. Khan et al. [50] showed that the drought-tolerant species of chickpea plant accumulated more proline than the drought-susceptible species. The previous findings have shown that when a plant experiences water stress, an increase in proline content is evident, with a protective role. On the other hand, increases in proline production could be evaluated as a response-to-protection mechanism [51,52]. It has been shown that proline contributes to the scavenging of reactive oxygen species (ROS). Our results indicated that higher accumulation of proline may be a strategy in drought-tolerant genotypes for maintaining the osmotic homeostasis. Previous studies showed that plants accumulating high amounts of proline also accumulated significant amounts of phenylalanine, one of the important amino acids playing crucial roles in the defense mechanism [47,53]. Thus, measuring proline along with phenylalanine might reveal how drought-tolerant plants also become disease-resistant.

The high lipid peroxidation in plants exposed to stress indicates that the plant has undergone oxidative damage, leading to the accumulation of MDA [54,55]. Lipid peroxidation is a marker of oxidative damage caused by both abiotic and biotic stresses [56]. The cell membrane becomes more damaged as the MDA concentration rises, and this is a widely used indicator to assess the response of plants [57]. It is evident that plants accumulating stress-related metabolites might show more tolerance to upcoming stress factors. Therefore, low MDA accumulating plants might have a greater impact on their tolerance against multiple stress factors.

After evaluation of various studies, it is evident that in the concept of the simultaneous drought-pathogen interactions, two significant stress factors stand out. For example, brown root rot (BRR) diseases caused by *Macrophomina phaseolina* and dry root rot (DRR) caused by *Fusarium solani* became more serious under severe drought stress conditions compared with the condition of irrigation in field conditions [26]. Similarly, findings were also made by other workers who stated that abiotic stress impacted wheat and barley plants to *Fusarium*-related diseases [58]. The plants became more vulnerable to those soil-borne pathogens.

This study highlighted that there would be a positive relation between *Fusarium* disease resistance and drought tolerance since *Foc*-resistant genotypes of chickpeas showed great tolerance to drought stress. We noticed that the disease-resistant or susceptible genotypes showed similar responses to drought stress; however, increased drought severity resulted in the accumulation of stress metabolites in disease-resistant plants. We found out that drought-tolerant chickpea plants were already disease-resistant to *Foc*. However, we need to further evaluate drought stress along with the pathogenic stress in different chickpea genotypes exhibiting drought stress tolerance or disease resistance to help plant breeders who would develop new varieties for sustainable agriculture.

## 5. Conclusions

Due to its high protein content, its importance increases as a significant alternate source of animal proteins. However, abiotic and biotic stressors reduce the quantity and quality of this plant. Recent studies have shown that stress factors combine and exert more devastating effects on chickpea cultivation. Although chickpea cultivation is quite challenging in a situation of increasing rainfall scarcity, adding pathogen stress to that condition could even create more complex conditions for plant breeders. Therefore, we need to breed more abiotic stress-tolerant and disease-resistant chickpea plants. Before this step, the mechanism of stress tolerance should be revealed in both separated and combined stressors. We could determine whether tolerance or resistance is characterized via a shared pathway in terms of biochemical and molecular mechanisms. If so, by triggering a common gene or genes, we could make chickpea plants tolerant and resistant to both stress factors. As an optimistic hope, we evaluated that Fusarium wilt and drought stresses are soil-borne and both stress factors affect the xylem vessel elements. Therefore, we consider that we could find and establish a logical solution for these complex stress interactions that would be more prevalent in the coming decades. Our studies regarding the biotic–abiotic stress responses of these chickpea varieties are underway.

The drought-susceptible genotype was clearly distinct from those of the above genotypes in terms of stress metabolite accumulations. Therefore, our next step is to use these genotypes under the combined stress of drought and *Foc*. If drought stress tolerance correlates with resistance to Fusarium infection, our job would be much easier to find common pathways generated by common genes. Work on the metabolic pathway is on our agenda, and we need to evaluate this mechanism in depth to breed more resistant and tolerant chickpea cultivars for drought and pathogenic responses.

## Figures and Tables

**Table 1:** GE (%), GRI, MGT, and VI of chickpea genotypes under different PEG6000 concentrations. Results were expressed as mean ± SE.

Seed Type	Genotype	PEG (%)	WP (MPa)	GP (%) *	GE	VI	GRI	MGT	Hypocotyl Height	Radicle Length
Desi	WR-315	0	0	85 ± 0.0 ^a1^	82.50 ± 2.5 ^a^	136.4 ± 20.3 ^bc^	6.49 ± 0.5 ^a^	5.25 ± 0.02 ^a^	0.075 ± 0.075 ^b^	1.50 ± 0.07 ^bc^
		5	−0.05	85 ± 0.0 ^a^	78.75 ± 6.9 ^a^	821 ± 265 ^a^	5.68 ± 0.4 ^a^	5.43 ± 0.23 ^a^	2.84 ± 1.11 ^a^	6.58 ± 1.85 ^a^
		7.5	−0.075	90 ± 5.77 ^a^	72.50 ± 6.6 ^a^	455 ± 42.7 ^ab^	5.49 ± 0.3 ^a^	5.36 ± 0.03 ^a^	1.055 ± 0.35 ^a^	2.90 ± 0.969 ^bc^
		10	−0.15	100 ± 0.0 ^a^	82.50 ± 3.2 ^a^	455 ± 4.12 ^ab^	6.62 ± 0.4 ^a^	5.23 ± 0.03 ^a^	0.975 ± 0.7 ^b^	3.57 ± 0.041 ^ab^
		15	−0.22	85 ± 5.0 ^a^	65.00 ± 8.4 ^a^	0 ± 0.0 ^c^	3.89 ± 0.4 ^b^	5.75 ± 0.08 ^a^	0 ± 0.0 ^b^	0 ± 0.0 ^c^
		20	−0.5	5 ± 5.0 ^b^	5.00 ± 0.0 ^b^	0 ± 0.0 ^c^	0.43 ± 0.2 ^c^	4.13 ± 1.66 ^a^	0 ± 0.0 ^b^	0 ± 0.0 ^c^
Desi	BG-212	0	0	40 ± 0.0 ^c^	28.75 ± 4.27 ^c^	16.8 ± 16.8 ^c^	1.29 ± 0.33 ^cd^	6.3 ± 0.30 ^a^	0.05 ± 0.05 ^c^	0.37 ± 0.37 ^d^
		5	−0.05	100 ± 0.0 ^a^	88.75 ± 3.75 ^a^	1584.5 ± 62.7 ^a^	5.46 ± 0.57 ^a^	5.59 ± 0.09 ^b^	4.36 ± 0.22 ^a^	11.48 ± 0.48 ^a^
		7.5	−0.075	85 ± 5.0 ^b^	68.75 ± 9.44 ^ab^	1072 ± 124 ^b^	3.02 ± 0.59 ^bc^	6.26 ± 0.18 ^ab^	1.86 ± 0.58 ^b^	10.64 ± 0.15 ^a^
		10	−0.15	100 ± 0.0 ^a^	83.75 ± 3.75 ^ab^	895 ± 11.8 ^b^	4.51 ± 0.52 ^ab^	5.78 ± 0.11 ^ab^	0.07 ± 0.07 ^c^	8.88 ± 0.58 ^b^
		15	−0.22	75 ± 5.0 ^b^	68.75 ± 3.75 ^ab^	126.5 ± 30.4 ^c^	3.49 ± 0.57 ^ab^	5.90 ± 0.13 ^ab^	0 ± 0.0 ^c^	1.75 ± 0.47 ^c^
		20	−0.5	75 ± 5.0 ^b^	63.75 ± 5.54 ^b^	5.60 ± 5.60 ^c^	3.63 ± 0.58 ^ab^	5.73 ± 0.14 ^ab^	0 ± 0.0 ^c^	0.07 ± 0.07 ^d^
Desi	JG-74	0	0	100 ± 0.0 ^a^	87.50 ± 5.2 ^ab^	1444 ± 164 ^a^	4.97 ± 0.29 ^b^	5.67 ± 0.10 ^ab^	4.46 ± 0.56 ^a^	9.97 ± 1.08 ^a^
		5	−0.05	100 ± 0.0 ^a^	90 ± 4.08 ^a^	884 ± 32.3 ^b^	5.17 ± 0.41 ^b^	5.69 ± 0.04 ^ab^	1.77 ± 0.22 ^b^	7.06 ± 0.20 ^b^
		7.5	−0.075	75 ± 5.77 ^b^	72.5 ± 6.61 ^b^	298 ± 11 ^cd^	4.41 ± 0.77 ^b^	5.66 ± 0.10 ^ab^	0.72 ± 0.41 ^bc^	3.73 ± 1.25 ^c^
		10	−0.15	100 ± 0.0 ^a^	88.75 ± 1.25 ^a^	546.5 ± 44.5 ^c^	7.09 ± 0.63 ^a^	5.22 ± 0.02 ^b^	0.59 ± 0.19 ^bc^	4.87 ± 0.28 ^bc^
		15	−0.22	75 ± 5.0 ^b^	17.50 ± 2.5 ^c^	5.6 ± 5.6 ^d^	0.80 ± 0.30 ^c^	6.37 ± 0.47 ^a^	0 ± 0.0 ^c^	0 ± 0.0 ^d^
		20	−0.5	75 ± 5.0 ^b^	0 ± 0.0 ^d^	0 ± 0.0 ^d^	0 ± 0.0 ^c^	0 ± 0.0 ^c^	0 ± 0.0 ^c^	0 ± 0.0 ^d^
Desi	CPS-1	0	0	75 ± 5.0 ^a^	61.25 ± 6.88 ^ab^	82.4 ± 36.1 ^bc^	3.79 ± 0.68 ^a^	5.79 ± 0.23 ^a^	0.07 ± 0.07 ^b^	0.95 ± 0.40 ^ab^
		5	−0.05	50 ± 5.77 ^b^	71.25 ± 6.25 ^a^	126.5 ± 73.3 ^bc^	3.61 ± 0.50 ^a^	5.92 ± 0.14 ^a^	0.56 ± 0.35 ^a^	1.54 ± 0.88 ^ab^
		7.5	−0.075	85 ± 5.00 ^a^	70 ± 7.91 ^a^	292.1 ± 28.4 ^a^	3.78 ± 0.49 ^a^	5.91 ± 0.14 ^a^	1.00 ± 0.10 ^a^	2.42 ± 0.14 ^a^
		10	−0.15	85 ± 5.00 ^a^	37.5 ± 5.20 ^c^	220.9 ± 24.9 ^ab^	2.53 ± 0.53 ^a^	5.66 ± 0.25 ^a^	0.18 ± 0.18 ^b^	2.42 ± 0.11 ^a^
		15	−0.22	50 ± 5.77 ^b^	46.25 ± 2.39 ^bc^	24.8 ± 18.1 ^c^	2.21 ± 0.28 ^a^	5.92 ± 0.08 ^a^	0 ± 0.0 ^b^	0.46 ± 0.30 ^ab^
		20	−0.5	0 ± 0.0 ^c^	0 ± 0.0 ^d^	0 ± 0.0 ^c^	0 ± 0.0 ^b^	0 ± 0.0 ^b^	0 ± 0.0 ^b^	0 ± 0.0 ^b^
Desi	K-850	0	0	90.0 ± 5.77 ^a^	81.25 ± 3.15 ^ab^	529.5 ± 51 ^a^	5.88 ± 0.30 ^ab^	5.37 ± 0.03 ^a^	-	6.39 ± 0.20 ^a^
		5	−0.05	100 ± 0.00 ^a^	93.75 ± 3.75 ^a^	639.5 ± 8.66 ^a^	7.19 ± 0.50 ^a^	5.29 ± 0.06 ^a^	-	5.61 ± 0.08 ^a^
		7.5	−0.075	95 ± 5.00 ^a^	81.25 ± 6.88 ^ab^	465 ± 118 ^a^	5.21 ± 0.62 ^b^	5.61 ± 0.09 ^a^	-	4.72 ± 1.11 ^a^
		10	−0.15	85 ± 5.00 ^a^	71.25 ± 7.74 ^b^	124.9 ± 51.8 ^b^	5.18 ± 0.56 ^b^	5.43 ± 0.11 ^a^	-	1.40 ± 0.51 ^b^
		15	−0.22	5 ± 5.00 ^b^	5.00 ± 0.0 ^c^	0 ± 0.0 ^b^	0.15 ± 0.15 ^c^	1.63 ± 0.11 ^b^	-	0.375 ± 0.375 ^b^
		20	−0.5	0 ± 0.0 ^b^	0 ± 0.0 ^c^	0 ± 0.0 ^b^	0 ± 0.0 ^c^	0 ± 0.0 ^b^	-	0 ± 0.0 ^b^
Desi	JG-62	0	0	95 ± 2.89 ^a^	86.25 ± 5.54 ^a^	1164 ± 93.9 ^a^	6.28 ± 0.45 ^bc^	5.38 ± 0.07 ^abc^	3.98 ± 0.78 ^a^	7.66 ± 0.19 ^a^
		5	−0.05	75 ± 2.89 ^b^	77.50 ± 9.68 ^a^	793 ± 80.8 ^b^	4.82 ± 0.49 ^c^	5.71 ± 0.11 ^ab^	1.90 ± 0.90 ^b^	8.0 ± 0.45 ^a^
		7.5	−0.075	80 ± 0.0 ^b^	91.25 ± 5.91 ^a^	683 ± 56.4 ^b^	7.06 ± 0.45 ^ab^	5.32 ± 0.06 ^bc^	1.13 ± 0.39 ^b^	7.40 ± 0.51 ^a^
		10	−0.15	75 ± 0.0 ^b^	95 ± 3.54 ^a^	473 ± 164 ^b^	8.58 ± 0.22 ^a^	5.07 ± 0.02 ^c^	0.74 ± 0.32 ^b^	3.99 ± 1.38 ^b^
		15	−0.22	40 ± 0.0 ^c^	33.75 ± 2.39 ^b^	51.3 ± 30.6 ^c^	1.98 ± 0.65 ^d^	5.78 ± 0.21 ^a^	0 ± 0.0 ^b^	1.28 ± 0.76 ^bc^
		20	−0.5	0 ± 0.0 ^d^	0 ± 0.0 ^c^	0 ± 0.0 ^c^	0 ± 0.0 ^e^	0 ± 0.0 ^d^	0 ± 0.0 ^b^	0 ± 0.0 ^c^
Desi	CHAFFA	0	0	95 ± 5.0 ^a^	83.75 ± 7.18 ^a^	1278.1 ± 95.4 ^a^	5.25 ± 0.46 ^a^	5.63 ± 0.06 ^a^	2.92 ± 0.14 ^a^	10.67 ± 1.23 ^a^
		5	−0.05	100 ± 0.0 ^a^	90 ± 5.40 ^a^	987 ± 96 ^b^	6.22 ± 0.62 ^a^	5.45 ± 0.10 ^a^	0.90 ± 0.53 ^b^	8.97 ± 0.78 ^ab^
		7.5	−0.075	100 ± 0.0 ^a^	88.75 ± 4.27 ^a^	852.5 ± 43.3 ^b^	6.96 ± 0.55 ^a^	5.27 ± 0.05 ^a^	0.80 ± 0.29 ^b^	7.72 ± 0.17 ^b^
		10	−0.15	95 ± 5.0 ^a^	88.75 ± 4.73 ^a^	784.5 ± 84.2 ^b^	6.35 ± 0.21 ^a^	5.39 ± 0.06 ^a^	0 ± 0.0 ^b^	8.21 ± 0.61 ^ab^
		15	−0.22	30 ± 5.77 ^b^	21.25 ± 2.39 ^b^	14.6 ± 14.6 ^c^	1.75 ± 0.69 ^b^	5.79 ± 0.64 ^a^	0 ± 0.0 ^b^	0.36 ± 0.36 ^c^
		20	−0.5	0 ± 0.0 ^c^	0 ± 0.0 ^c^	0 ± 0.0 ^c^	0 ± 0.0 ^b^	0 ± 0.0 ^b^	0 ± 0.0 ^b^	0 ± 0.0 ^c^
Desi	BG-215	0	0	100 ± 0.0 ^a^	87.50 ± 5.20 ^ab^	1633 ± 128 ^a^	6.72 ± 0.37 ^a^	5.38 ± 0.05 ^b^	4.13 ± 0.67 ^a^	12.19 ± 0.80 ^a^
		5	−0.05	95 ± 5.0 ^a^	90 ± 4.08 ^a^	1084 ± 193 ^b^	6.21 ± 0.34 ^a^	5.40 ± 0.03 ^b^	1.66 ± 0.36 ^b^	9.70 ± 1.41 ^ab^
		7.5	−0.075	95 ± 5.0 ^a^	72.50 ± 6.61 ^b^	916 ± 132 ^b^	4.33 ± 0.45 ^b^	5.75 ± 0.14 ^a^	1.60 ± 0.09 ^b^	7.89 ± 1.05 ^b^
		10	−0.15	95 ± 5.0 ^a^	88.75 ± 1.25 ^a^	243 ± 124 ^c^	6.53 ± 0.51 ^a^	5.30 ± 0.04 ^b^	1.00 ± 0.54 ^bc^	1.42 ± 0.71 ^c^
		15	−0.22	65 ± 5.0 ^b^	17.50 ± 2.50 ^c^	0 ± 0.0 ^c^	4.60 ± 0.12 ^b^	5.33 ± 0.07 ^b^	0 ± 0.0 ^c^	0 ± 0.0 ^c^
		20	−0.5	0 ± 0.0 ^c^	0 ± 0.0 ^d^	0 ± 0.0 ^c^	0 ± 0.0 ^c^	0 ± 0.0 ^c^	0 ± 0.0 ^c^	0 ± 0.0 ^c^
Desi	ANNIGERI	0	0	55 ± 5.0 ^c^	50 ± 3.54 ^b^	75.1 ± 28.1 ^b^	2.78 ± 0.27 ^b^	5.75 ± 0.22 ^a^	0.07 ± 0.07 ^b^	1.31 ± 0.44 ^b^
		5	−0.05	70 ± 5.77 ^bc^	67.50 ± 8.54 ^ab^	593.5 ± 22.1 ^a^	3.83 ± 0.88 ^ab^	5.97 ± 0.23 ^a^	1.74 ± 0.65 ^a^	6.89 ± 0.66 ^a^
		7.5	−0.075	100 ± 0.0 ^a^	76.25 ± 5.91 ^a^	600 ± 206 ^a^	5.36 ± 0.25 ^a^	5.43 ± 0.03 ^a^	0.99 ± 0.42 ^ab^	5.0 ± 1.70 ^a^
		10	−0.15	80 ± 8.16 ^b^	62.50 ± 5.95 ^ab^	153.7 ± 60.9 ^b^	4.97 ± 0.93 ^a^	5.36 ± 0.22 ^a^	0.24 ± 0.13 ^b^	1.80 ± 0.63 ^b^
		15	−0.22	10 ± 5.77 ^d^	7.50 ± 1.44 ^c^	4.50 ± 4.50 ^b^	0.37 ± 0.29 ^c^	3.25 ± 1.92 ^a^	0 ± 0.0 ^b^	0.22 ± 0.22 ^b^
		20	−0.5	0 ± 0.0 ^d^	0 ± 0.0 ^c^	0 ± 0.0 ^b^	0 ± 0.0 ^c^	0 ± 0.0 ^b^	0 ± 0.0 ^b^	0 ± 0.0 ^b^
Kabuli	C-104	0	0	100 ± 0.0 ^a^	87.50 ± 5.20 ^a^	904.5 ± 31.5 ^a^	5.97 ± 0.39 ^a^	5.46 ± 0.05 ^b^	0.66 ± 0.23 ^a^	8.38 ± 0.23 ^a^
		5	−0.05	75 ± 2.04 ^b^	65 ± 7.91 ^b^	669 ± 51 ^b^	3.31 ± 0.41 ^b^	5.97 ± 0.12 ^a^	0.44 ± 0.17 ^a^	7.91 ± 0.78 ^a^
		7.5	−0.075	70 ± 2.04 ^c^	67.50 ± 5.20 ^b^	499.6 ± 91.3 ^b^	4.17 ± 0.47 ^b^	5.64 ± 0.10 ^b^	0 ± 0.0 ^b^	6.25 ± 1.14 ^a^
		10	−0.15	0 ± 0.0 ^d^	0 ± 0.0 ^c^	0 ± 0.0 ^c^	0 ± 0.0 ^c^	0 ± 0.0 ^c^	0 ± 0.0 ^b^	0 ± 0.0 ^b^
		15	−0.22	0 ± 0.0 ^d^	0 ± 0.0 ^c^	0 ± 0.0 ^c^	0 ± 0.0 ^c^	0 ± 0.0 ^c^	0 ± 0.0 ^b^	0 ± 0.0 ^b^
		20	−0.5	0 ± 0.0 ^d^	0 ± 0.0 ^c^	0 ± 0.0 ^c^	0 ± 0.0 ^c^	0 ± 0.0 ^c^	0 ± 0.0 ^b^	0 ± 0.0 ^b^
Kabuli	UC-27	0	0	85 ± 5.0 ^a^	75 ± 4.56 ^a^	147.7 ± 57.6 ^a^	5.52 ± 0.33 ^a^	5.38 ± 0.05 ^a^	-	1.708 ± 0.67 ^a^
		5	−0.05	65 ± 9.57 ^ab^	60 ± 2.89 ^b^	69 ± 69 ^ab^	4.28 ± 0.63 ^ab^	5.48 ± 0.09 ^a^	-	0.863 ± 0.86 ^a^
		7.5	−0.075	45 ± 9.57 ^bc^	41.25 ± 2.39 ^c^	0 ± 0.0 ^b^	3.01 ± 0.22 ^b^	5.35 ± 0.10 ^a^	-	0 ± 0.0 ^a^
		10	−0.15	35 ± 9.57 ^cd^	30 ± 3.54 ^d^	0 ± 0.0 ^b^	0.44 ± 0.27 ^c^	6.08 ± 0.33 ^a^	-	0 ± 0.0 ^a^
		15	−0.22	15 ± 5.0 ^de^	12.50 ± 1.44 ^e^	0 ± 0.0 ^b^	0 ± 0.0 ^c^	3.23 ± 1.87 ^a^	-	0 ± 0.0 ^a^
		20	−0.5	0 ± 0.0 ^e^	0 ± 0.0 ^f^	0 ± 0.0 ^b^	0 ± 0.0 ^c^	0 ± 0.0 ^b^	-	0 ± 0.0 ^a^
Kabuli	ILC-482	0	0	65 ± 5.0 ^a^	58.75 ± 3.15 ^ab^	8 ± 8.0 ^a^	3.69 ± 0.46 ^ab^	5.60 ± 0.11 ^a^	0 ± 0.0 ^b^	0.10 ± 0.10 ^a^
		5	−0.05	60 ± 8.16 ^a^	47.50 ± 6.61 ^b^	244 ± 150 ^a^	2.65 ± 0.39 ^bc^	5.87 ± 0.15 ^a^	1.87 ± 0.93 ^a^	1.49 ± 0.92 ^a^
		7.5	−0.075	60 ± 0.0 ^a^	51.25 ± 5.91 ^ab^	189 ± 81.3 ^a^	2.90 ± 0.40 ^bc^	5.81 ± 0.18 ^a^	0.37 ± 0.37 ^ab^	2.78 ± 1.22 ^a^
		10	−0.15	70 ± 5.77 ^a^	65 ± 2.04 ^a^	172 ± 121 ^a^	4.64 ± 0.44 ^a^	5.38 ± 0.05 ^a^	0.26 ± 0.26 ^ab^	1.91 ± 1.25 ^a^
		15	−0.22	30 ± 5.77 ^b^	25 ± 2.89 ^c^	0 ± 0.0 ^a^	1.62 ± 0.54 ^c^	5.78 ± 0.30 ^a^	0 ± 0.0 ^b^	0 ± 0.0 ^a^
		20	−0.5	0 ± 0.0 ^c^	0 ± 0.0 ^d^	0 ± 0.0 ^a^	0 ± 0.0 ^d^	0 ± 0.0 ^b^	0 ± 0.0 ^b^	0 ± 0.0 ^a^

* GP: Germination percentage (%) GE: Germination energy; VI: Vigor index; GRI: Germination rate index; MGT: Mean germination time; ^1^: Different letters in columns show significant differences *p* < 0.05 level.

**Table 2:** Pathological and physiological responses of chickpea genotypes to *Fusarium oxysporum* f. sp. *ciceris* races [34,35], and drought responses on the basis of IC_50_ values.

Seed Type	Genotypes	IC_50_	Race0	Race1A	Race2	Race4	Race5
**Desi**	WR-315	13.93 ^a^	R	R	R	R	R
**Desi**	BG-212	17.76 ^a^	R	R	S	M	R
**Desi**	JG-74	16.83 ^a^	R	R	S	R	M
**Desi**	CPS-1	10.04 ^b^	R	R	S	M	M
**Desi**	K-850	11.49 ^b^	-	-			
**Desi**	JG-62	11.54 ^b^	R	S	S	S	S
**Desi**	CHAFFA	15.24 ^a^		S	R	S	
**Desi**	BG-215	13.93 ^a^					
**Desi**	Annigeri	10.13 ^b^		R	S	S	
**Kabuli**	C-104	7.38 ^c^	R	M	S	S	S
**Kabuli**	UC-27	6.95 ^c^			M	M	
**Kabuli**	ILC-482	8.53 ^c^					

R: Resistant, S: Susceptible, M: Moderately susceptible. Different letters in columns show significant differences *p* < 0.05 level.

## Data Availability

The data that support the findings of this study are available from the corresponding author.

## References

[1] Gaur P.M., Samineni S., Sajja S., Chibbar R.N. (2015). Achievements and Challenges in Improving Nutritional Quality of Chickpea. Legume Perspect..

[2] Alcorta A., Porta A., Amparo T., Dolores Alvarez M., Pilar Vaquero M. (2021). Foods for Plant-Based Diets: Challenges and Innovations Alexandra. Foods.

[3] Gopalakrishnan S., Srinivas V., Samineni S. (2017). Nitrogen Fixation, Plant Growth and Yield Enhancements by Diazotrophic Growth-Promoting Bacteria in Two Cultivars of Chickpea (*Cicer arietinum* L.). Biocatal. Agric. Biotechnol..

[4] Tiwari S., Sahu V.K., Gupta N., Tripathi M.K., Yasin M. (2020). Evaluation of Physiological and Biochemical Contents in Desi and Kabuli Chickpea. Legume Res. Int. J..

[5] Arora N.K. (2019). Impact of Climate Change on Agriculture Production and Its Sustainable Solutions. Environ. Sustain..

[6] Koskosidis A., Khah E., Mavromatis A., Pavli O., Vlachostergios D.N. (2020). Effect of PEG-Induced Drought Stress on Germination of Ten Chickpea (*Cicer arietinum* L.) Genotypes. Not. Bot. Horti Agrobot. Cluj-Napoca.

[7] Devasirvatham V., Tan D.K.Y. (2018). Impact of High Temperature and Drought Stresses on Chickpea Production. Agronomy.

[8] Song L., Jin J., He J. (2019). Effects of Severe Water Stress on Maize Growth Processes in the Field. Sustainability.

[9] Reed R.C., Bradford K.J., Khanday I. (2022). Seed Germination and Vigor: Ensuring Crop Sustainability in a Changing Climate. Heredity.

[10] Yousefi A.R., Rashidi S., Moradi P., Mastinu A. (2020). Germination and Seedling Growth Responses of *Zygophyllum fabago*, *Salsola kali* L. and *Atriplex canescens* to Peg-Induced Drought Stress. Environments.

[11] Domozych D.S., Kozel L., Palacio-Lopez K. (2021). The Effects of Osmotic Stress on the Cell Wall-Plasma Membrane Domains of the Unicellular Streptophyte, *Penium margaritaceum*. Protoplasma.

[12] Kalefetoǧlu Macar T., Turan Ö., Ekmekçi Y. (2009). Effects of Water Deficit Induced by PEG and NaCl on Chickpea (*Cicer arietinum* L.) Cultivars and Lines at Early Seedling Stages. Gazi Univ. J. Sci..

[13] Nikitin D.A., Ivanova E.A., Semenov M.V., Zhelezova A.D., Ksenofontova N.A., Tkhakakhova A.K., Kholodov V.A. (2023). Diversity, Ecological Characteristics and Identification of Some Problematic Phytopathogenic Fusarium in Soil: A Review. Diversity.

[14] Kheiri A., Moosawi Jorf S.A., Malihipour A. (2019). Infection Process and Wheat Response to Fusarium Head Blight Caused by Fusarium Graminearum. Eur. J. Plant Pathol..

[15] Bhar A., Jain A., Das S. (2021). Soil Pathogen, *Fusarium oxysporum* Induced Wilt Disease in Chickpea: A Review on Its Dynamicity and Possible Control Strategies. Proc. Indian Natl. Sci. Acad..

[16] Dikilitas M., Karakas S. (2014). Crop Plants under Saline-Adapted Fungal Pathogens. Emerging Technologies and Management of Crop Stress Tolerance.

[17] Baran B., Ölmez F., Çapa B., Dikilitas M. (2024). Defense Pathways of Wheat Plants Inoculated with *Zymoseptoria tritici* under NaCl Stress Conditions: An Overview. Life.

[18] Dubey S.C., Priyanka K., Singh V., Singh B. (2012). Race Profiling and Molecular Diversity Analysis of *Fusarium oxysporum* f. Sp. *ciceris* Causing Wilt in Chickpea. J. Phytopathol..

[19] Kocalar H., Kafadar F.N., Ozkan A., Talapov T., Demirel O., Anay A., Mart D., Can C. (2020). Current Distribution and Virulence of *Fusarium oxysporum* f. Sp. *Ciceris* in Turkey. Legume Res..

[20] Jiménez-Gasco M.D.M., Milgroom M.G., Jiménez-Díaz R.M. (2004). Stepwise Evolution of Races in *Fusarium oxysporum* f. Sp. *ciceris* Inferred from Fingerprinting with Repetitive DNA Sequences. Phytopathology.

[21] Muche M., Yemata G. (2022). Epidemiology and Pathogenicity of Vascular Wilt of Chickpea (*Cicer arietinum* L.) Caused by *Fusarium oxysporum* f. Sp. *ciceris*, and the Host Defense Responses. S. Afr. J. Bot..

[22] Trapero-Casas A., Jimenez-Diaz R.M. (1985). Fungal Wilt and Root Rot Diseases of Chickpea in Southern Spain. Phytopathology.

[23] Haware M.P., Nene Y.L. (1982). Races of *Fusarium oxysporum* f. Sp. *ciceri*. Plant Dis..

[24] Dikilitas M., Karakas S., Ashraf M., Öztürk M., Ahmad M.S.A., Aksoy A. (2012). Behaviour of Plant Pathogens for Crops Under Stress During the Determination of Physiological, Biochemical, and Molecular Approaches for Salt Stress Tolerance.

[25] Dikilitas M., Karakas S., Hashem A., Abd Allah E.F., Ahmad P. (2016). Oxidative Stress and Plant Responses to Pathogens under Drought Conditions. Water Stress Crop Plants Sustain. Approach.

[26] Sinha R., Irulappan V., Mohan-Raju B., Suganthi A., Senthil-Kumar M. (2019). Impact of Drought Stress on Simultaneously Occurring Pathogen Infection in Field-Grown Chickpea. Sci. Rep..

[27] Michel B.E., Kaufmann M.R. (1973). The Osmotic Potential of Polyethylene Glycol 6000. Plant Physiol..

[28] Ullah A., Sadaf S., Ullah S., Alshaya H., Okla M.K. (2022). Using Halothermal Time Model to Describe Barley (*Hordeumvulgare* L.) Seed Germination Response to Water Potential and Temperature. Life.

[29] Itroutwar P.D., Kasivelu G., Raguraman V., Malaichamy K., Sevathapandian S.K. (2020). Effects of Biogenic Zinc Oxide Nanoparticles on Seed Germination and Seedling Vigor of Maize (*Zea mays*). Biocatal. Agric. Biotechnol..

[30] Bakhshandeh E., Jamali M., Afshoon E., Gholamhossieni M. (2017). Using Hydrothermal Time Concept to Describe Sesame (*Sesamum indicum* L.) Seed Germination Response to Temperature and Water Potential. Acta Physiol. Plant..

[31] Carlson J.R., Ditterline R.L., Martin J.M., Sands D.C., Lund R.E. (1983). Alfalfa Seed Germination in Antiobiotic Agar Containing NaCl. Crop Sci..

[32] Dikilitas M. (2003). Effect of Salinity & Its Interactions with Verticillium Albo-Atrum on the Disease Development in Tomato (*Lycopersicon esculentum* Mill.) and Lucerne (*Medicago sativa* L. & M. media) Plants. Ph.D. Thesis.

[33] Bates L.S., Waldren R.P., Teare I.D. (1973). Rapid Determination of Free Proline for Water—Stress Studies. Plant Soil.

[34] Heath R.L., Packer L. (1968). Photoperoxidation in Isolated Chloroplasts. II. Role of Electron Transfer. Arch. Biochem. Biophys..

[35] Dolar F.S. (1997). Determination of the Races of *Fusarium oxysporum* f. Sp. *ciceris* in Ankara Province, Turkey. J. Turk. Phytopqthology.

[36] Jiménez-Díaz R.M., Castillo P., del Mar Jiménez-Gasco M., Landa B.B., Navas-Cortés J.A. (2015). Fusarium Wilt of Chickpeas: Biology, Ecology and Management. Crop Prot..

[37] Farooq M., Ullah A., Lee D.J., Alghamdi S.S., Siddique K.H.M. (2018). Desi Chickpea Genotypes Tolerate Drought Stress Better than Kabuli Types by Modulating Germination Metabolism, Trehalose Accumulation, and Carbon Assimilation. Plant Physiol. Biochem..

[38] Wahab A., Abdi G., Saleem M.H., Ali B., Ullah S., Shah W., Mumtaz S., Yasin G., Muresan C.C., Marc R.A. (2022). Alleviate the Adverse Effects of Drought Stress: A Comprehensive Review. Plants.

[39] Seleiman M.F., Al-Suhaibani N., Ali N., Akmal M., Alotaibi M., Refay Y., Dindaroglu T., Abdul-Wajid H.H., Battaglia M.L. (2021). Drought Stress Impacts on Plants and Different Approaches to Alleviate Its Adverse Effects. Plants.

[40] Ahmad M.A., Javed R., Adeel M., Rizwan M., Yang Y. (2020). PEG 6000-Stimulated Drought Stress Improves the Attributes of in Vitro Growth, Steviol Glycosides Production, and Antioxidant Activities in Stevia Rebaudiana Bertoni. Plants.

[41] Adetunji A.E., Sershen, Varghese B., Pammenter N.W. (2020). Effects of Inorganic Salt Solutions on Vigour, Viability, Oxidative Metabolism and Germination Enzymes in Aged Cabbage and Lettuce Seeds. Plants.

[42] Himaja R., Radhika K., Reddy K.B., Raghavendra M. (2021). Screening of Chickpea (Cicer Arietinum L.) Genotypes for Germination and Early Seedling Growth under PEG 6000 Induced Drought Stress. Legume Res..

[43] Abderemane B.A., Houasli C., Mitache M., Idrissi O., Fakiri M. (2024). Physiological, Agro-Morphological, and Germination Responses of a Worldwide Chickpea (*Cicer arietinum*) Collection Subjected to Drought Stress by Applying Polyethylene Glycol (PEG) on Germinating Seeds and by Exposure Plants to Water Restriction at the Vegetative Stage. Biocatal. Agric. Biotechnol..

[44] Haghpanah M., Hashemipetroudi S., Arzani A., Araniti F. (2024). Drought Tolerance in Plants: Physiological and Molecular Responses. Plants.

[45] Terfa G.N., Pan W., Hu L., Hao J., Zhao Q., Jia Y., Nie X. (2025). Mechanisms of Salt and Drought Stress Responses in Foxtail Millet. Plants.

[46] Wen J., Shi J., Meng M., Xu K., Xu Y., Ji D., Wang W., Xie C. (2025). Metabolic Responses of *Pyropia haitanensis* to Dehydration-Rehydration Cycles Revealed by Metabolomics. Mar. Drugs.

[47] Adamipour N., Nazari F., Nalousi A.M., Teixeira Da Silva J.A. (2025). Evaluation of the Molecular Mechanism Underlying Proline Metabolic and Catabolic Pathways and Some Morpho-Physiological Traits of Tobacco (*Nicotiana tabacum* L.) Plants under Arsenic Stress. BMC Plant Biol.

[48] Gazi R., Kumar S., Jana M. (2025). Proline Concentration Driven Thermostability and Hydration Properties of Ubiquitin. J. Mol. Liq..

[49] Yan L., Lu M., Riaz M., Tong K., Yu H., Gao G., Niu Y. (2025). Exogenous Proline Enhances Salt Acclimation in Soybean Seedlings: Modifying Physicochemical Properties and Controlling Proline Metabolism through the Ornithine-Glutamate Dual Pathway. Ecotoxicol. Environ. Saf..

[50] Khan N., Bano A., Rahman M.A., Guo J., Kang Z., Babar M.A. (2019). Comparative Physiological and Metabolic Analysis Reveals a Complex Mechanism Involved in Drought Tolerance in Chickpea (*Cicer arietinum* L.) Induced by PGPR and PGRs. Sci. Rep..

[51] Furlan A.L., Bianucci E., Giordano W., Castro S., Becker D.F. (2020). Proline Metabolic Dynamics and Implications in Drought Tolerance of Peanut Plants. Plant Physiol. Biochem..

[52] Desoky E.S.M., Mansour E., El-Sobky E.S.E.A., Abdul-Hamid M.I., Taha T.F., Elakkad H.A., Arnaout S.M.A.I., Eid R.S.M., El-Tarabily K.A., Yasin M.A.T. (2021). Physio-Biochemical and Agronomic Responses of Faba Beans to Exogenously Applied Nano-Silicon Under Drought Stress Conditions. Front. Plant Sci..

[53] Machado J., Fernandes A.P.G., Bokor B., Vaculík M., Kostoláni D., Kokavcová A., Heuvelink E., Vasconcelos M.W., Carvalho S.M.P. (2025). Tomato Responses to Nitrogen, Drought and Combined Stresses: Shared and Specific Effects on Vascular Plant Anatomy, Nutrient Partitioning and Amino Acids Profile. Plant Physiol. Biochem..

[54] Martinez V., Mestre T.C., Rubio F., Girones-Vilaplana A., Moreno D.A., Mittler R., Rivero R.M. (2016). Accumulation of Flavonols over Hydroxycinnamic Acids Favors Oxidative Damage Protection under Abiotic Stress. Front. Plant Sci..

[55] Khaleghi A., Naderi R., Brunetti C., Maserti B.E., Salami S.A., Babalar M. (2019). Morphological, Physiochemical and Antioxidant Responses of Maclura Pomifera to Drought Stress. Sci. Rep..

[56] Sarker U., Oba S. (2020). The Response of Salinity Stress-Induced A. Tricolor to Growth, Anatomy, Physiology, Non-Enzymatic and Enzymatic Antioxidants. Front. Plant Sci..

[57] Zhao H., Guan J., Liang Q., Zhang X., Hu H., Zhang J. (2021). Effects of Cadmium Stress on Growth and Physiological Characteristics of Sassafras Seedlings. Sci. Rep..

[58] Martínez M., Arata A.F., Lázaro L., Stenglein S.A., Dinolfo M.I. (2019). Effects of Waterlogging Stress on Plant-Pathogen Interaction between *Fusarium poae* and Wheat/Barley. Acta Sci. Agron..

